# Topical drug delivery strategies for enhancing drug effectiveness by skin barriers, drug delivery systems and individualized dosing

**DOI:** 10.3389/fphar.2023.1333986

**Published:** 2024-01-16

**Authors:** Lin Zhao, Jiamei Chen, Bai Bai, Guili Song, Jingwen Zhang, Han Yu, Shiwei Huang, Zhang Wang, Guanghua Lu

**Affiliations:** ^1^ State Key Laboratory of Southwestern Chinese Medicine Resources, Chengdu University of Traditional Chinese Medicine, Chengdu, China; ^2^ School of Ethnic Medicine, Chengdu University of Traditional Chinese Medicine, Chengdu, China; ^3^ School of Pharmacy, Chengdu University of Traditional Chinese Medicine, Chengdu, China

**Keywords:** topical drug, drug effectiveness, skin barriers, individualized dosing, drug delivery

## Abstract

Topical drug delivery is widely used in various diseases because of the advantages of not passing through the gastrointestinal tract, avoiding gastrointestinal irritation and hepatic first-pass effect, and reaching the lesion directly to reduce unnecessary adverse reactions. The skin helps the organism to defend itself against a huge majority of external aggressions and is one of the most important lines of defense of the body. However, the skin’s strong barrier ability is also a huge obstacle to the effectiveness of topical medications. Allowing the bioactive, composition in a drug to pass through the stratum corneum barrier as needed to reach the target site is the most essential need for the bioactive, composition to exert its therapeutic effect. The state of the skin barrier, the choice of delivery system for the bioactive, composition, and individualized disease detection and dosing planning influence the effectiveness of topical medications. Nowadays, enhancing transdermal absorption of topically applied drugs is the hottest research area. However, enhancing transdermal absorption of drugs is not the first choice to improve the effectiveness of all drugs. Excessive transdermal absorption enhances topical drug accumulation at non-target sites and the occurrence of adverse reactions. This paper introduces topical drug delivery strategies to improve drug effectiveness from three perspectives: skin barrier, drug delivery system and individualized drug delivery, describes the current status and shortcomings of topical drug research, and provides new directions and ideas for topical drug research.

## 1 Introduction

**FIGURE 1:**
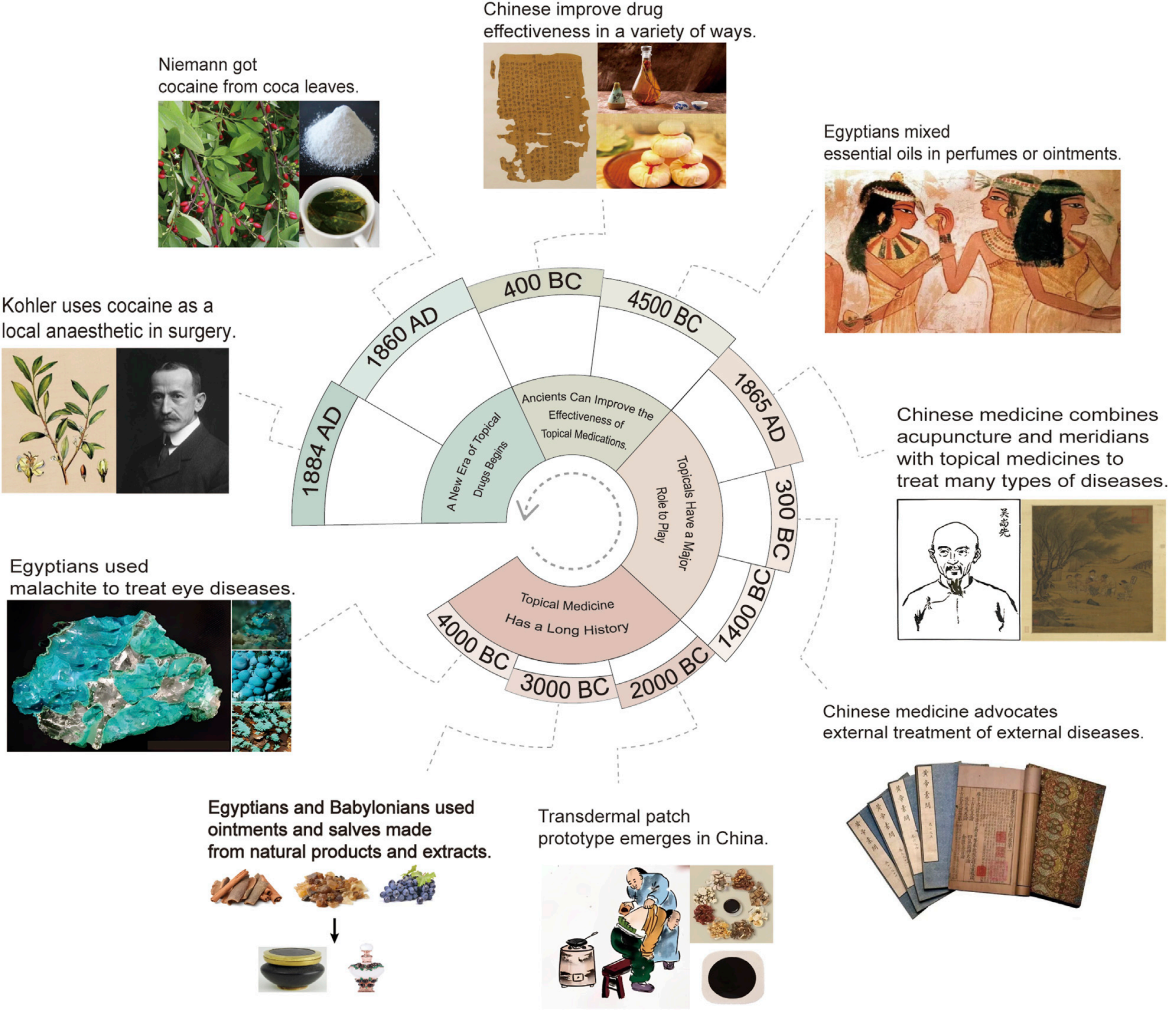
Development process of traditional topical drugs. The development process is divided into four aspects: topical medicine has a long history, topical drugs play an important role, the ancients can improve the efficacy of topical drugs, and topical drugs have begun a new era.

Topical drugs have a long history. Thousands of years ago, ointments and salves made from animal, mineral, or plant extracts were commonly used in Egyptians, Chinese and Babylonians to cure a wide range of ailments (Lima et al., 2021; Metwaly et al., 2021; Roberts et al., 2021). Before 2000 BC, emplastra appeared in China, which maybe the original transdermal patch (Pastore et al., 2015). Many topical drugs are so effective that they are now widely used in many countries, and are also the source of discovery of some very effective monomers. However, there are some topical drugs that seem somewhat incomprehensible nowadays. For example, Kahun gynecological papyrus records that a substance (possibly crocodile excrement) is treated with honey or kefir and injected into the vagina for contraception (Metwaly et al., 2021). During the period of using natural drugs to treat diseases, external medication is one of the most important means of treating diseases. Surviving frescoes and books attest to this (Pastore et al., 2015; Chen, 2016; Zheng and Zheng, 2017). In terms of enhancing the effectiveness of topical drugs, many attempts have been made and many useful results have been achieved. Ancient Egyptians added essential oils to perfumes or ointments to increase the transdermal absorption efficiency of the active ingredients (Elshafie and Camale, 2017); Chinese used wine to soak their medicines to realize the enrichment of the active ingredients and to increase the transdermal absorption efficiency of the active ingredients (Zhu, 2007); and Chinese also heated up topical medicines added with iron sand to be used after application, which is a kind of topical drug formulation design to enhance the therapeutic efficacy of the medicines by heating (Zhu, 2007). In 1860, German chemist Niemann extracted an alkaloid from coca leaves and named it as cocaine. This is the first small molecule topical drug that is generally recognized. In 1884, cocaine was first officially used as a local anesthetic in clinical practice (Grzybowski, 2008). Since then, small molecule drugs have become the overlord of topical drugs to replace natural medicines (Figure 1).

With the continuous development of science and technology and people’s deepening of disease understanding, peptides, proteins, nucleic acids, cells and bacteria are gradually used as drugs. The ultimate goal of all topical medication is to reach the target site *in vivo* with enough dose without side effects. According to the different target sites, topical medication can be divided into four types: 1) Drugs that do not want to be absorbed (e.g., sunscreen, heavy metals); 2) Drugs that reach the skin tissue and do not want to spread (e.g., drugs for the treatment of skin diseases); 3) Drugs that reach deeper tissues (e.g., anesthetics, drugs for treating muscle or joint diseases); 4) Drugs that cross the skin into blood vessels and are transported to other tissues or organs (e.g., insulin). In this paper, diseases are divided into three categories according to different target sites of disease (Table 1).

**TABLE 1:** Classification of diseases at different target sites for topical drugs.

Target site	Disease
Skin	Skin Photoaging, Wrinkles, Skin pores are enlarged openings, Pigmentation diseases, Melasma, Congenital giant nevi, Pathogenesis of Port-Wine Stains, Hand Eczema, Atopic Dermatitis, psoriasis, Ichthyosis, Inflammation of actinic keratoses, Seborrheic keratoses, Localized scleroderma, Dermatitis linearis, Phytophotodermatitis, Contact Hypersensitivity Reactions, Acute generalized exanthematous pustulosis, Seborrheic dermatitis, Radiodermatitis, Hidradenitis Suppurativa, Cutaneous leishmaniasis, Pittosporum folliculitis, Acne Vulgaris, Rosacea, Cutaneous warts, Molluscum contagiosum, Human monkeypox virus, Dermatophytomas, Nonmelanoma skin cancers, Melanoma, Vitiligo, Bullous pemphigoid, Uberous Sclerosis Complex, Hyperhidrosis, Lupus erythematosus, Female exual function, Severe phimosis, Cold injuries, Burns, Scarring, Triae cutis distensae, Calcinosis cutis, Buruli ulcer, Peyronie’s disease, Vulvar Paget’s disease, Clitoral Keratin Pearls, Bowen’s disease, Pyoderma gangrenosum, Hypergranulation, Androgenic Alopecia
Subcutaneous tissue	Anaesthesia, Wound Healing, Pain, Chronic pruritus, Painful diabetic peripheral neuropathy, Treatment of chemotherapy-induced peripheral neuropathy, Acute soft tissues injuries, Psoriatic arthritis of the temporomandibular joint, Rheumatoid arthritis, Erectile dysfunction, Postoperative seroma, Surgical site infections, Diabetic foot infections, Diabetic foot ulcers, Hronic venous ulcerations, Idiopathic granulomatous mastitis, Varicose Ulcers, Erosive pustular dermatosis, Early breast cancer, Mycosis Fungoides-Type Cutaneous T-Cell Lymphoma, Facial angiofibromas, Muscle function, Microcystic lymphatic malformation, Minimal Bruising, Osteoarthritis, Recurrent lymphangiopathy of the external urethra, Blood loss in patients undergoing prosthetic knee surgery, Gorlin-Goltz syndrome
Other tissues and organs	Slimming, Chronic Constipation, Cognitive impairment, Insomnia symptoms, Depression, Osteoporotic, Sjögren’s Syndrome, Graft-versus-host disease, Diabetes

## 2 Properties and functions of four different skin barriers

**FIGURE 2:**
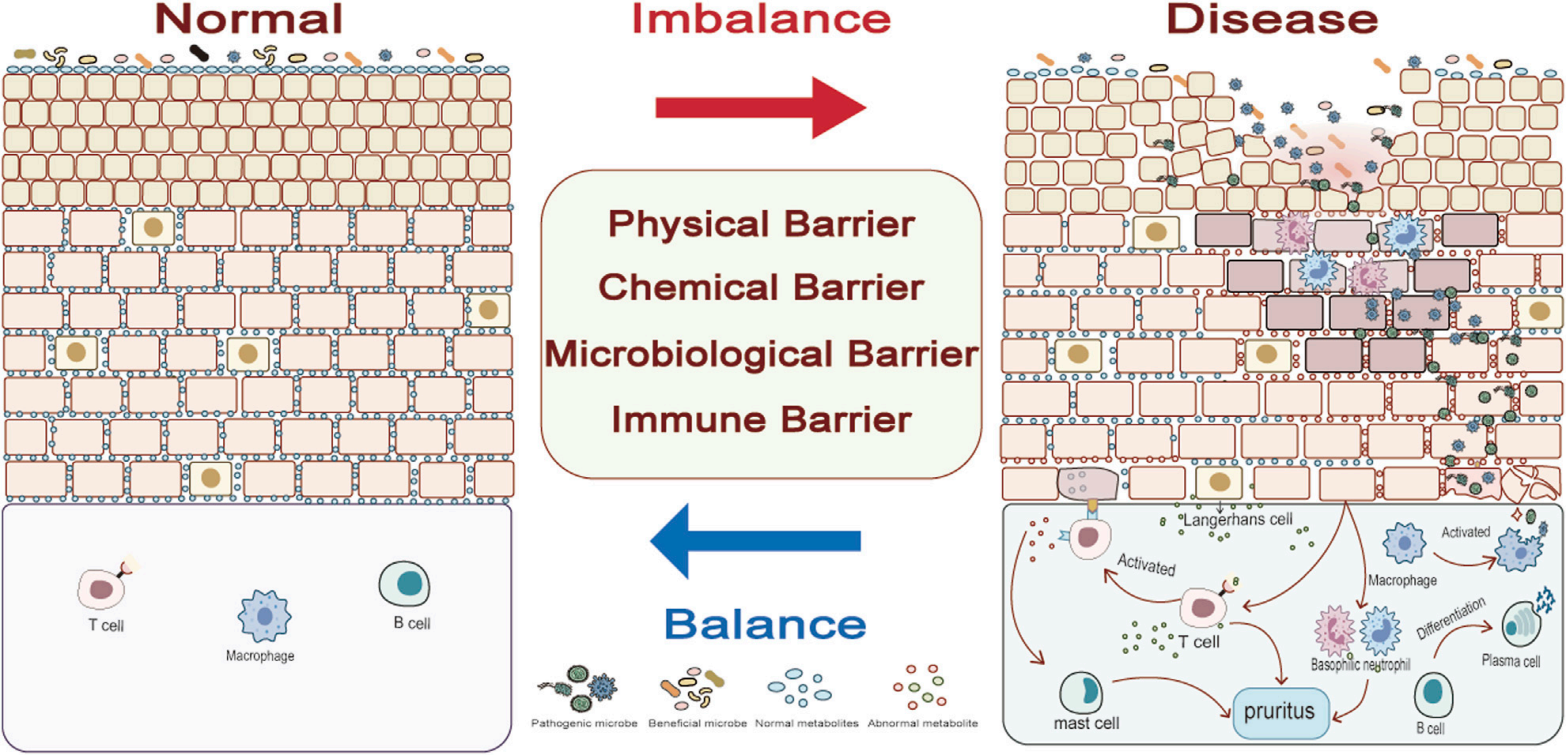
The balance of the four skin barriers is the key to healthy skin. The four skin barriers are closely related and do not exist in isolation. Abnormalities in the microbiological and physical barriers are often the beginning of disease, and their abnormalities cause changes in the immune and chemical barriers. Only when all four barriers are restored to balance will the skin return to normal.

Recently, our view of the skin has evolved from a mere physical barrier to an organic organ made up of microbial, chemical, physical and immune barriers (Eyerich et al., 2018). Microbial, physical, chemical and immune barriers work synergistically together to maintain homeostasis in the body (Figure 2).

The microbial barrier consists of bacteria, archaea, fungi and viruses which inhabit the surface of the skin and mucous membranes. The microbial barrier can maintain and enhance the skin barrier function through a variety of mechanisms such as competing for nutrients, secreting metabolites, and interfering with the quorum sensing system, inhibiting conditional pathogenic bacterial infection (Uberoi et al., 2021), regulating immunity (Dhariwala and Scharschmidt, 2021), and promoting cell and tissue regeneration (Wang et al., 2023). For example, *Staphylococcus* epidermidis can affect immune cells in a number of ways thereby promoting neonatal skin barrier development (Severn and Horswill, 2023), enhancing innate immunity and inhibiting invasion by pathogenic bacteria (Gallo, 2015). Balancing the microbial barrier is key to maintaining healthy skin. Not only can increased abundance of pathogenic bacteria cause or exacerbate disease, but also can non-pathogenic bacteria (Severn and Horswill, 2023). And pathogenic bacteria are not entirely harmful. Under homeostatic conditions, the immune response induced by *Staphylococcus aureus* colonization of skin tissue can promote the growth of damaged nerve axons and local nerve regeneration (Enamorado et al., 2023).

The physical barrier is made up of the cells that make up the structure of the skin and is divided into epidermis, dermis and subcutaneous tissue. They balance body temperature and moisture, protect the body from ultraviolet exposure, transmit sensations and maintain good health (Eyerich et al., 2018). The stratum corneum is the outermost layer of the epidermis, with a thickness of about 10–20 μm, high density, low water content, low surface area of solute transport, and inactive metabolism, which is figuratively likened to a “brick wall structure” (Yang et al., 2017). The epidermis is mainly composed of keratinocytes with different degrees of differentiation. The keratinocytes move upwards from the basal layer, the degree of differentiation increases, the cells continue to age until they die, and the dead cells eventually accumulate in the outermost layer to form the stratum corneum (Peskoller et al., 2022). The dermis is located below the epidermis, and the basal layer is connected to the dermis layer by means of a basement membrane, which is mainly composed of fibroblasts and plays a supporting role in the epidermis (Ogawa, 2017). Cushioning the epidermis and dermis are layers of subcutaneous tissue and fat that protect the body from damage, provide flexibility and strength, act as a barrier to infection, and act as heat insulation and shock absorption (Singh and Singh, 1993). There are also skin appendages such as hair follicles, sebaceous glands, sweat glands, and nails on the surface of the skin, many of which are directly connected to the dermis and are an important way for drugs, especially macromolecular drugs, to enter the blood. Hair follicles are highly conserved sensory organs that are important reservoirs of keratinocytes and melanocytes in the skin, and hair follicles are also implicated in immune response to pathogens, thermoregulation, sebum production, angiogenesis, neurogenesis and wound healing (Ji et al., 2021). Sebaceous gland is a component of the sebaceous gland unit of the hair follicle, which secretes sebum, and its state is related to hair follicle morphogenesis (Zouboulis et al., 2022). The human body has about 20,000 to 40,000 sweat glands, which are divided into apocrine sweat glands and eccrine sweat glands according to anatomical structure and location (Sato et al., 1989). The end of the exocrine sweat duct is on the surface of the skin, and the end of the apocrine sweat duct is in the hair follicle.

The chemical barrier consists of metabolites from skin cells and microorganisms, and the chemical composition of the barrier varies from site to site, but they all play an important role in maintaining the balance of the skin barrier. The epidermis contains ceramides, free fatty acids, cholesterol, cholesterol sulphates, cholesterol esters and other lipids that are important for maintaining the barrier function of the skin (Starr et al., 2022). The research shows that the skin barrier function is significantly reduced after stratum corneum lipid extraction (Sweeney and Downing, 1970). The main components in sebum are mainly composed of glycerol ester, free fatty acid, cholesterol, Cholesteryl ester, wax ester and marlene. They are skin lubricants and stratum corneum plasticizing lipids, which can maintain the acidic condition of the outer surface of the skin and enhance the skin barrier function, and also play a central role in the composition and functional regulation of the skin microbiome (Qiu et al., 2022; Yin et al., 2023). Dysregulation of sebum secretion is an crucial cause or exacerbation of inflammatory skin disease (Shi et al., 2015). Amino acids, sodium pyrrolidone carboxylate, lactate, urea and other composition with moisturizing functions produced by the disintegration of keratinocytes are called natural moisturizing factors. Natural moisturizing factors are mainly distributed in the stratum corneum, which can regulate the hydration function of keratinocytes, maintain normal skin permeability, and reduce percutaneous water loss of the skin (Mojumdar et al., 2017). The metabolites of microorganisms mainly play a regulatory role. For example, sphingomyelinase can increase ceramide content in the stratum corneum, reduce skin dehydration, and enhance skin barrier integrity (Zheng et al., 2022). Short-chain fatty acids can regulate inflammation and promote wound healing (Stacy and Belkaid, 2019). Sweat glands producing hypotonic solutions can regulation and skin hydration (Roberts et al., 2021). These hypotonic solutions, together with other substances on the surface of the skin, constitute hundreds of components of sweat, and these components can reflect the state of the body (Yang et al., 2023). There is growing evidence that sweat can become a new type of substance to be tested, which can be used to detect diseases or special conditions such as alcohol or drug use.

The immune barrier is an inflammation immune regulatory system composed of various immune cells and inflammatory molecules in the skin. Under normal circumstances, only a small percentage of immune cells reside between the skin tissues. These immune cells residing among skin tissues can respond immediately when the skin is attacked by external forces, such as Langerhans cell and memory cells, which maintain skin stability by secreting antibodies, splitting cells, phagocytosis of pathogens and other means (Kabashima et al., 2019). Microorganisms are an important source for the immune barrier to respond appropriately to stimuli. The moist is an excellent place for microorganisms to communicate with the immune system (Chen et al., 2018). The skin is composed of microorganisms, cells, and the chemicals they secrete. It is an organic whole, and changes in every link can cause skin diseases. The cross-era development of biotechnology has focused research on the single-cell level, and breakthroughs in the analysis of very small samples of nucleic acids, proteins, small molecule compounds and other components have allowed us to deepen our understanding of the occurrence and development of diseases. Cell activity in disease states is summarized here, as detailed in Table 2. Understanding the dynamic changes of different cells in the occurrence and development of diseases is conducive to patients to choose more suitable drugs in different disease states, and improve effectiveness, increase the cure rate of patients, and reduce the pain of patients by actively avoiding drugs with poor effects, or through some measures to improve the efficiency of drugs reaching the target site.

**TABLE 2:** The role of different cells in skin diseases.

Target cells	Brief introduction	Disease	Function	Ref
Keratinocytes	Keratinocytes are the main cell types in the living cortex, the vast majority of the living cortex undergoes complete renewal approximately every 28 days, but only 15 percent of cells are continuously involved in this process	Psoriasis	1. Excessive proliferation and abnormal differentiation cause infiltration of a variety of inflammatory cells	Wickett and Visscher. (2006), Proksch et al. (2008), Baroni et al. (2012), Thyssen et al. (2013), Chieosilapatham et al. (2021), Flori et al. (2022), Zhou et al. (2022)
			2. Releases its own nucleotides and antimicrobial peptides, activating plasmacyte-like dendritic cells	
			3. Together with fibroblasts and endothelial cells, tissue remodeling occurs through the activation and proliferation of endothelial cells and the deposition of extracellular matrix	
		Atopic dermatitis	Cytokines such as TSLP, interleukin-25, and interleukin-33 are released, and dendritic cells, mast cells, basophils, and T cells are activated to produce or aggravate inflammatory dermal infiltration and itching	
		Ichthyosis	Loss of filagin gene function leads to cytoskeletal disorders, altered lamellar cargo loading, and impaired lamellar maturation, resulting in dryness, hyperkeratosis, excessive desquamation, keratosis pilaris, and palmar and plantar hyperlinearity	
		Chloasma	Overexpression of paracrine melanin-producing factor	
Melanocytes	Melanocyte originate from pigment particles (particles containing melanin produced by the Neural crest are called melanosomes), which have an important function of protecting the skin from ultraviolet radiation and giving color to the skin, hair and eyes	Alopecia areata	Under external stimuli, apoptotic melanocytes debris induce an immune response and cause alopecia areata	Jimbow et al. (1986), Barrera et al., 1995, Xie et al. (2022), Oliveira et al. (2022), Yi et al. (2023)
		Melanoma	1. Melanoma cells amplify CD4 Treg mainly through major histocompatibility complex-II, thereby promoting immune erosion and accelerating immune progress. 2. Sirtuin7 of Melanoma cells is activated by unsaturated polyester resins, which promotes cell survival and immune escape and accelerates the progress of Melanoma	
Sebaceous cells	Sebocytes are located in a unique “small resistance site” within the dermis and play an important role in preserving and balancing the symbiotic environment of lipids, cytokines, and resident bacteria of the sebaceous unit of hair follicles	Chloasma	Cytokines (pro-melanogenic factorsα-melanocyte-stimulating hormone, Human Endothelin-1; the stromal sustaining factors Stemcellfactor and Basic Fibroblast Growth Factor) and release of lipid molecules, Promotes melanin production and intensifies pro-inflammatory cytokines-mediated inflammatory responses	Zouboulis et al. (2022), Flori et al. (2022)
Fibroblasts	Fibroblasts are synthetic and remodeling extracellular matrices in embryonic and adult organs and are the main cellular responsible for tissue and organ types of fibrosis, skin scarring, atherosclerosis, systemic sclerosis, and the formation of atherosclerotic plaques following vascular damage. In the dermis, fibroblasts are its main cell type. According to their spatial location, they can be divided into many subtypes. For example, fibroblast-like fibroblasts in the dermis layer adjacent to the basement membrane and epidermis, reticular fibroblasts located in the lower reticular dermis. Different subtypes also have different functions. Fibroblasts have a tendency to fibrosis and can regulate the growth and differentiation of epidermal keratinocytes. Reticulofibroblasts secrete most of the fine extracellular matrix in the dermis, responsible for the first wave of dermal repair after full-thickness wounds. Dermal fibroblasts, located in the dermis and connective tissue sheaths, have the ability to regulate the signaling required for hair follicle morphogenesis and hair growth coordination	Keloids	Caused by fibroblast hyperplasia and abnormal accumulation of extracellular matrix such as collagen	Rinkevich et al. (2015), Ghetti et al. (2018), Kim et al. (2019), Alicea et al. (2020), Hughes et al. (2020), Deng et al. (2021), Tsou et al. (2021), O’Neill et al. (2022), Gur et al. (2022), Flori et al. (2022), Xu et al. (2022)
		Chloasma	Overexpression of paracrine melanin-producing factor in fibroblasts, especially aged fibroblasts	
		Systemic sclerosis	1. Quiescent fibroblasts are converted into myofibroblasts, which promotes the expression of α-smooth muscle actin, the secretion of matrix proteins, and the increase of stress fibers	
			2. The decrease in the number and dysfunction of fibroblasts associated with Scleroderma cause fibrosis, vascular pathology, inflammation*etc.*	
		Inflammatory skin diseases	Reduced expression of latent transforming growth factor beta binding protein 4, IGFBP5, and TCF4 in fibroblasts in inflammatory skin diseases	
		Psoriasis	C-C chemokine ligand 19, TNF superfamily member 13b and Chemokine Ligand 12 expression were elevated	
		Acne	Fibroblasts can undergo local proliferation and differentiation into a pre-adipocyte’s lineage, secreting the antimicrobial peptide cathelicidin, which inhibits bacterial growth. At the same time, the large amount of oil produced during this reaction can promote the growth of bacteria	
		Melanoma	Lipids secreted by aging skin fibroblasts enhance melanoma resistance to targeted drugs	
		Vitiligo	Dermal fibroblasts recruit more CD8^+^ T cells in response to interferon γ secreted by cytotoxic CD8^+^ T cells and secrete the chemokines Chemokine ligand 9 and Recombinant Human C-X-C Motif Chemokine 10, further expanding the progression of vitiligo	
Merkel’s cell	Merkle cells are closely related to nerve endings and function as sensory receptors in the nervous system	alloknesis	The epidermal Piezo2 channel-Merkle cell signaling regulates the transition from touch to pruritus and participates in the exodus neuromechanoreceptors that stimulate the process of conduction from the skin to the spinal cord	Wickett and Visscher. (2006), Feng et al. (2018)
Neutrophils	Neutrophils are the most abundant immune cells in humans and are key players in the acute immune response with a short half-life. Normally, neutrophils are released from the bone marrow into the bloodstream and circulate in the body for less than a day. It plays a central role in the body’s defense against the onset and continuation of external microbial and systemic autoimmune diseases, such as dysregulation of neutrophil activation that can lead to tissue damage during various diseases	Atopic dermatitis	1. Basophils accumulate at the site of the lesion	Byrd et al. (2019), Lopez-Sanz et al. (2021), Wang et al. (2021), Dudeck et al. (2021), Shibuya et al. (2021), Wigerblad and Kaplan (2023)
			2. Basophil secretionIL33, IL4, TSLP, IL31 and other cytokines cause itching	
		Allergic inflammation	Basophils cause dysregulated Th2 immune response	
		Hidradenitis suppurativa	1. Neutrophil infiltration, enhancing spontaneous extracellular traps	
			2. Promote B cell activation and autoantibody secretion, causing immune dysregulation and leading to inflammation	
Monocyte	Monocytes originate from progenitor cells in the bone marrow and are transported through the bloodstream to peripheral tissues. Regulated by local growth factors, pro-inflammatory cytokines, and microbial products, they differentiate into macrophages or dendritic cell populations. Monocytes contribute greatly to the effective control and elimination of viral, bacterial, fungal and protozoan infections, but also promote the development of inflammatory and degenerative diseases	Wound	Monocytes act as rheostats during wound repair by regulating leptin levels and vascular reconstruction	Shi and Pamer. (2011), Lum et al. (2022), Kratofil et al. (2022)
		Bacterial infections	Monocytes are transformed into macrophages, which regulate the expansion of subcutaneous adipocytes and the production of the adipokine hormone leptin for several weeks after infection	
		Monkeypox	Infection of Monocyte will accelerate the systemic infection of Monkeypox virus	
Congenital lymphocytes	Innate lymphoid cells are a heterogeneous group of immune cells characterized by lymphoid morphology and cytokine profiles, similar to T cells but not expressing antigen receptors, which play an important role in innate immunity. Innate lymphoid cells are a heterogeneous group of immune cells characterized by lymphoid morphology and cytokine profiles, similar to T cells, but do not express antigen receptors, and play an important role in innate immunity. According to cytokines, transcription factors and functions, it is divided into three groups, the first group is composed of ILC1 and natural killer; The second group consisted of ILC2; The third group consists of ILC3 and Long-Term immunosuppression	Psoriasis	ILC3 specifically produces the associated cytokines IL-17: and IL-22 in response to IL-23 signaling, leading to skin inflammation	Kobayashi et al. (2020), Bielecki et al. (2021), Yu et al. (2022)
Mast cell	Mast cells are innate immune cells that end-differentiate and reside within vascular tissue and often play a key role in the initiation and continuation of allergic inflammation, often through IgE-mediated mechanisms. As sentinels of local tissues, mast cells play a vital role in the host’s defense against certain parasites, bacteria, and toxins. Enclosed inside mast cells is a dense set of secretory granules containing soluble mediators, including vasoactive amines and proteases. And, activated mast cells exhibit a remarkable ability to synthesize and release multiple chemokines, cytokines, lipid-derived mediators, and antimicrobial peptides	Chloasma	Mast cells increase significantly	Lopez-Sanz et al. (2021), Wang et al. (2021), Falduto et al. (2021), Dudeck et al. (2021), Shibuya et al. (2021), Flori et al. (2022)
		Atopic dermatitis	1. Aggregation at the site of the lesion	
			2. Secretion of IL33, IL4, TSLP, IL31 and other cytokines causes itching	
		Inflammation	Mast cell-derived TNF activates endothelial cells within blood vessels. Neutrophils circulating in the blood are directly activated in order to migrate to inflamed tissues	
		Allergic inflammation	Causes dysregulation of the Th2 immune response	
Dendritic cells	Dendritic cells, a class of bone marrow-derived cells found in blood, epithelial, and lymphoid tissue, are key players in mediating immune responses and inducing immune tolerance. Dendritic cells have molecular sensors and antigen processing mechanisms that can recognize pathogens, integrate chemical information, and guide immune responses, including multiple subtypes such as plasma celloid dendritic cells, CD14 dendritic cells+, Langerhans cells, and inflammatory dendritic cells [76]. Among them, Langerhans cells are the epitome of dendritic cells of migrating tissues, which not only participate in antigen presentation, but also play an important role in controlling the proliferation of keratinocytes, and are essential for the epidermal immune barrier	Cutaneous lupus	1. Secretion of pro-inflammatory cytokines, chemokines, and co-stimulatory molecules continuously activates innate and adaptive immune responses	Potten and Allen. (1976), Pieri et al. (2001), Cumberbatch et al. (2003), Luessi et al. (2016), Macri et al. (2018), Liu et al. (2020), Hughes et al. (2020), Karnell et al. (2021), Tsou et al. (2021), Liu et al. (2021), Lum et al. (2022)
		Systemic sclerosis	1.Plasmacytic-like dendritic cells produce interferon to promote the occurrence of disease	
			2. Upregulate the interaction of CCL5-CCR3, TNFSF9-TNFRSF9, XCL2-XCR1 and other receptors/ligands	
		Inflammatory skin diseases	1. Recombinant Interleukin 1 Receptor Type I, Recombinant Interleukin 1 Receptor Type II and C-C chemokine receptor type 7, as well as Fc receptor expression	
			2. Recruitment and polarization of T cells	
		Psoriasis	LC2 is elevated in skin lesions of psoriasis, expressing immunomodulatory markers	
		Monkeypox	Dendritic cell macrophage infection will accelerate the systemic infection of Monkeypox virus	
Innate lymphoid cells	Intrinsic lymphoid cells are a population of immune cells that do not express rearranged antigen receptors and are mainly enriched on the barrier surface, mainly by producing cytokines to coordinate immune response, promote barrier integrity and maintain tissue homeostasis. According to the expression of lineage-specific transcription factors, native lymphoid cells can be divided into ILC1, ILC2 and ILC3, and natural killer cells belong to ILC1	leprosy	Increased expression of IDO1, STAT1, HCAR3, and MHC class I molecules	Hughes et al. (2020), Zhou et al. (2022)
Macrophage	Macrophages are highly plastic cells with a variety of functions, including tissue development and homeostasis, removal of cellular debris, elimination of pathogens, and regulation of inflammatory responses. Macrophage activation states are generally simplified into two categories: M1 classically activated macrophages and M2 replacement activated macrophages	Inflammatory skin disease	Expression of CD163, STAB1 and CEPP increased	Hughes et al. (2020), Anderson et al. (2021), Lum et al. (2022), Do et al. (2022)
		Acne	Increased macrophage triggering receptor expressed on myeloid cells 2 subsets adjacent to hair follicles and sebaceous glands	
		Monkeypox	Infection with macrophages accelerates systemic infection with monkeypox virus	
T cells	T cells are key players in maintaining immune homeostasis. After exposure to the pathogen, T lymphocytes migrate to the barrier site, providing local immunity and long-term protection to the body. After the pathogen disappears, the human barrier tissue still contains tissue resident Memory T cell, which show different tissue site specificity of residence, homing and function from circulating Memory T cell	Atopic dermatitis	1. Regulatory T cell dysfunction imbalance, antigen processing and presentation, apoptosis and other immune responses and the number of various types of T cells increased significantly at the lesion site	Gaydosik et al. (2021), Wang et al. (2021), Tsou et al. (2021), Huang et al. (2021), Wegrzyn et al. (2022), Zhang et al. (2022), Lum et al. (2022), Oliveira et al. (2022), Humeau et al. (2022), Xu et al. (2022), Liu et al. (2022), Poon et al. (2023), Hasegawa et al. (2023)
			2. Th2 cells secrete IL33, IL4, TSLP, IL31 and other cytokines to cause itching	
		Senescence	Cytotoxic CD4^+^ T cells can help fight aging by clearing senescent cells	
		Systemic sclerosis	1. Increased subsets of profibrotic T cells	
			2. Th17 and Th22 cell drives involve inflammatory responses in fibroblasts and endothelial cells	
			3. CD4^+^ T cells decreased methylation of CD40L and CD11a, and CD70 demethylation and corresponding expression were enhanced, which promoted the occurrence of inflammatory response and induced T cells to migrate to fibrosis	
		Rheumatoid arthritis	When chronic inflammation occurs, abnormal CpG methylation occurs in the cis-regulatory region of the gene, resulting in the loss of regulatory T cell function, further causing abnormal regulation of effector T cells, aggravating the disease	
		Tumor	CD4 T cells can promote or suppress the antitumor response by recognizing antigens presented by human leukocyte antigen class II molecules	
		Melanoma	CD8-CCR7 and CD8-NR4A1 are involved in the positive regulation of lymphocyte activation, cell response to heat, and cell response to tumor necrosis factor	
		Hair loss	Regulatory T cells can sense changes in glucocorticoid levels at the hair loss site, release TGF-β3, activate the proliferation of hair follicle stem cells, and the hair follicle enters the growth phase from the telogen phase	
		Vitiligo	Autoimmune CD8^+^ T cells attack epidermal melanocytes, causing depigmentated patches of skin	
		Monkeypox	1. T follicular helper cells enhance the recall and differentiation of memory B cells into antibody-secreting cells	
			2. Cytolytic T cells help kill infected macrophages to prevent the spread of the virus	
			3. CD8 T cells have been shown to eradicate virus-infected monocytes	
B cells	B cells have the unique ability to produce antibodies against multiple targets, providing protection against infection while also contributing to pathogenesis in an immunodysregulated environment. In addition to this, subsets of B cell functional specialization also promote immune responses through antigen presentation and cytokine production	Hidradenitis suppurativa	B cell dysregulation, such as elevated plasma cells and immunoglobulin G, promotes immune dysregulation and leads to inflammation	Byrd et al. (2019), Glass et al. (2020), Lum et al. (2022)
		Monkeypox	1. B cell infection will accelerate the systemic infection of Monkeypox virus	
			2. Vaccinia immunoglobulin secreted by B cells can significantly inhibit further infection with monkeypox virus	

## 3 Drug delivery systems and factors affecting transdermal drug absorption

**FIGURE 3:**
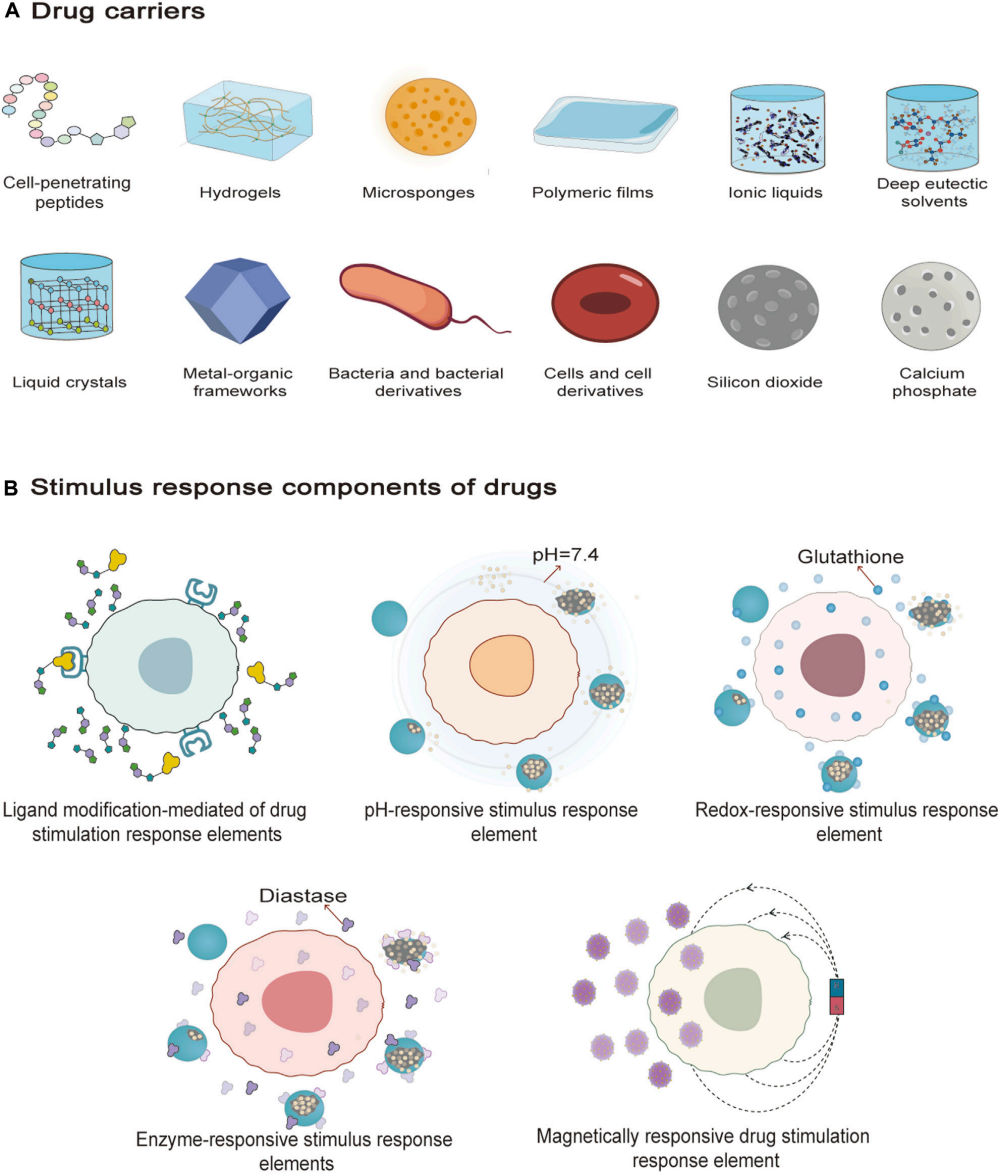
Drug carriers and drug stimulus response elements. **(A)** is the 12 drug carriers described in the text, which are cell-penetrating peptides, hydrogels, microsponges, polymer films, ionic liquids, deep eutectic solvents, liquid crystals, metal-organic frameworks, bacteria and bacterial derivatives, cells and cell derivatives, silicon dioxide, and calcium carbonate. **(B)** is the stimulus response element for five drugs described in the text, namely, ligand-mediated targeting of drug-smart response elements, pH-responsive smart response element, redox-responsive smart response element, enzyme-responsive smart response element, and magnetically-responsive drug smart response element.

Drug delivery system refers to a technological system that comprehensively regulates the distribution of drugs in an organism in terms of space, time and dose (Baryakova et al., 2023). Ideally, the drug delivery system should allow accumulation of the bioactive composition in the target site at the appropriate dose without overpenetration resulting in the drug reaching the circulation at a toxic dose or accumulating in tissues at non-target sites causing adverse effects (Peralta et al., 2018). Physicochemical properties of drug bioactive composition, excipients and additives, such as molecular weight, partition coefficient, polarity, surface charge and degree of hydrogen bonding, affect the absorption efficiency of drug bioactive composition (Mohammed et al., 2022). Selection of appropriate dosage forms, carriers, additives, and some smart response elements can improve the problems of poor stability, low solubility, and inability to accumulate at the target site of the drug bioactive composition (Figure 3). Garg et al. have given a very detailed account of the commonly used topical dosage forms and additives in their article. So this paper do not cover these two aspects (Garg et al., 2015).

### 3.1 Drug carriers

When the bioactive composition of a drug is poorly stabilized, low solubility, dose-limiting side effects, a narrow therapeutic window or short half-life that makes it difficult to maintain the proper concentration of the drug over a period of time, the choice of the right drug delivery system is an important way to address these issues. Ramanunny et al. described 16 drug delivery systems (Ramanunny et al., 2021). This paper builds on them with a further account of 16 drug carriers (Table 3).

**TABLE 3:** Advantages, disadvantages and applications of drug carriers.

Drug carriers	Advantages	Disadvantages	Applications	Ref
Cell-penetrating Peptides	Cell-penetrating peptides are highly permeable, low cytotoxic and non-immunoreactive	Cell-penetrating peptides are less stable, less specific and the drug has to cross the cytosol to act	Cell-penetrating peptides can be used as drug carriers for skin cancer and inflammatory skin diseases and as cosmetic carriers	Menegatti et al. (2016), Gan et al. (2018), Ruseska and Zimmer (2020)
Hydrogels	Hydrogels has a high drug loading capacity for sustained drug release	The disadvantages of hydrogels are related to the materials and production methods, such as hyaluronic acid material made of hydrogel mechanical strength is weak, low degradation rate; physical cross-linking method of hydrogel is susceptible to interference by external factors	Hydrogels are used as carriers for glucose, adriamycin, hyaluronic acid derivatives, dexamethasone and other drugs	Fonder et al. (2007), Narayanaswamy and Torchilin (2019), Patel et al. (2024)
Microsponges	Microsponges have high drug loading capacity, high biosafety, long residence time in the skin, reduced number of administration times, improved bioavailability of bioactive ingredients and facilitated transdermal absorption	Poor homogeneity, reproducibility, and transdermal ability of the microsponge	Microsponges can be used as carriers for topical sunscreens, RNA drugs, dermatological drugs, gene therapy, bone repair, and bioimaging	Grabow and Jaeger. (2012), Shopsowitz et al. (2014), Junqueira and Bruschi, (2018), Choi et al. (2019), Bhuptani and Patravale, (2019), Mahant et al. (2020); Zhang et al. (2021)
Polymeric films	Polymer films are small in diameter, highly porous and amorphous, and can act as a physical barrier by attaching to tissue or mucous membranes, providing physical support for the tissue, and delivering a quantitative, sustained release of drug to the tissue or mucous membrane	Adhesion of the polymer film to the skin or mucous membranes may cause discomfort, and the adhesive in the polymer film may cause allergies	Polymeric films can be used as an alternative to occlusive ointments and gauze as carriers for topical medications such as propranolol and betamethasone	Frederiksen et al. (2015), Hissae Yassue-Cordeiro et al. (2019), Padula et al. (2019), Thakkar et al. (2019), Liu et al. (2022), Riccio et al. (2022)
Ionic Liquids	Ionic liquids are safe, less irritating and promote transdermal absorption of topical drugs	The variety of ionic liquids is limited, there are fewer biosafety studies, and the aggregation properties of liquid formulations need to be studied in depth	Ionic liquids can be used as skin penetration enhancers, drug carriers, as dermatomimetic smart ionic gels with good antimicrobial effects, and have the potential to be used as RNA stabilisers	Mazid et al. (2014), Zakrewsky et al. (2014), Monti et al. (2017), Qian et al. (2017), Li et al. (2020), Correia et al. (2021), Zhang et al. (2023)
Deep Eutectic Solvents	Deep eutectic solvents have low toxicity and modulate the solubility, permeability and absorption of biologically active pharmaceutical ingredients	Deep eutectic solvents are less well studied with unclear principles and safety	Deep eutectic solvents can be used as stabilisers for protein components and to modify the properties of some carriers	Xin et al. (2017), Trombino et al. (2022), Ge et al. (2023)
Liquid Crystals	Liquid crystals offer improved drug loading, stability and controlled drug release as well as high bioadhesion and biocompatibility	Liquid crystal production and testing costs are high, and the crystal shape is susceptible to environmental influences	Liquid crystals can be made into breathable, shrinkable haemostatic patches	Wu et al. (2021), Chavda et al. (2023)
Metal-organic Frameworks	Metal-organic frameworks have controlled surface chemistry, high specific surface area, high porosity and reversible structural flexibility, high cellular uptake and good biocompatibility	Many metal ions are biotoxic	Metal-organic frameworks can be used as carriers for photodynamic therapy photosensitisers, anticancer drugs, antimicrobials, gaseous drugs and deep tissue imaging	Sun et al. (2020), Zhang et al. (2020), Luo et al. (2021), Cheng et al. (2022), Huang et al. (2022), Liu et al. (2022), Pan et al. (2022)
Bacteria	Bacteria have the ability to preferentially colonise and immunologically activate tumours	Bacterial production at scale, maintenance of bacterial viability during delivery, precise control of bacterial colonisation, dosimetry and biosafety are still major challenges	Bacteria can be used as carriers for tumour-targeting drugs, RNA vaccines, drugs to treat skin-based diseases	Gurbatri et al. (2020), Li et al. (2021), Pei et al. (2022), Liu et al. (2023), Lloren et al. (2023)
Bacterial Ghosts	Bacterial ghosts have bioadhesive properties and are also capable of stimulating an innate immune response without posing any threat of infection	The weak transdermal ability of bacterial ghosts often needs to be used in conjunction with microneedling and other means	Bacterial ghosts have important applications in adjuvants, vaccines and targeted drug carriers	Kudela et al. (2010), Hjelm et al. (2015), Michalek et al. (2017), Li et al. (2021), Shen et al. (2022)
Bacterial Extracellular Vesicles	Bacterial extracellular vesicles activate innate immunity	Methods for isolating and purifying bacterial extracellular vesicles have not been standardised and storage conditions for bacterial extracellular vesicles are harsh	Bacterial extracellular vesicles can directly treat dermatological diseases as well as serve as carriers for the delivery of RNA drugs, oncology drugs, macromolecular drugs, and drugs for the treatment of bacterial infections	Gurung et al. (2011), Jun et al. (2017), Huang et al. (2020), Li et al. (2022), Liu et al. (2023), Liu et al. (2023)
Cells	Cells are characterized by good biocompatibility, biodegradability and long half-life, which can solve the problems of premature clearance, toxicity and immunogenicity of synthetic nanocarriers	Cells are costly and ethically questionable to use	Cells are used directly as drugs to treat diseases and also as carriers for nanomedicines, targeted drugs for brain tumours, bone marrow-targeted drugs	Choi et al. (2021), Li et al. (2022), Chang et al. (2023)
Extracellular Vesicles	Extracellular vesicles are small, biocompatible, biodegradable, have a long half-life time, are very easily absorbed into the skin, and can solve the problems of premature clearance, toxicity and immunogenicity of synthetic nanocarriers without ethical issues	Extracellular vesicles are difficult to store and difficult to produce industrially	Extracellular vesicles can be used to directly treat a variety of diseases also as drug carriers for the delivery of RNA drugs, hyaluronic acid	Ma et al. (2021), Bui et al. (2022), Oh et al. (2022), Ma et al. (2023), You et al. (2023)
Cell Membrane	Cell membranes are biocompatible, biodegradable, have a long half-life, are easy to prepare, and can solve the problems of premature clearance, toxicity and immunogenicity of synthetic nanocarriers without ethical issues	Cell membranes are costly to produce and difficult to store	Cell membranes can be used as carriers for gene therapy, other nanomedicines and targeted drugs	Gao et al. (2020), Cao et al. (2022), Chen et al. (2022)
Silicon Dioxide	Silicon dioxide has a high specific surface area, good pore size tunability, and can be loaded with multiple active ingredients simultaneously to prolong administration time and reduce concentration	Silicon dioxide cytotoxicity, genotoxicity, *in vivo* metabolic pathway and other studies are less, need to follow up in-depth research	Silicon dioxide can be used as vectors for RNA drugs	Shen et al. (2014), Mora-Raimundo et al. (2021), Zhou et al. (2022)
Calcium Carbonate	Calcium carbonate can be loaded with a variety of drugs at the same time, and the calcium ions produced by decomposition under characteristically acidic conditions can regulate a wide range of physiological activities	Calcium carbonate is less researched, and there is a lack of clarity on a variety of issues such as the stability of the product being made and storage conditions	Calcium carbonate is used as a drug to activate channels associated with calcium ions and can be used as carriers for pH responsive components, a variety of drugs and photosensitizers	Zhu et al. (2020), Shen et al. (2022), Xu et al. (2022)

#### 3.1.1 Cell-penetrating peptides

Cell-penetrating peptides, also known as protein translocation structural domains, membrane translocation sequences, or trojan peptides, are small molecules of 6–30 amino acid residues that are able to penetrate biological barriers and cell membranes to enter the cell in a noninvasive manner (Silva et al., 2019). In 1988, Frankel et al. found that the tat protein of human immunodeficiency virus I was taken up into the nucleus by cells grown in tissue cultures (Frankel and Pabo, 1988). Since then, people have begun to take notice of cell-penetrating peptides. Over the course of more than 30 years, the family of cell-penetrating peptides has grown, with more than 100 cell-penetrating peptides described in articles by Milletti alone (Milletti, 2012). Cell-penetrating peptides are classified in a variety of ways. According to their physicochemical properties, cell-penetrating peptides can be simply divided into three categories: cationic, amphipathic and hydrophobic (Raucher and Ryu, 2015). The lack of cell type specificity of cell-penetrating peptides is one of the main reasons preventing their widespread use (Bode Lowik, 2017). The mechanism by which cell-penetrating peptides enter the cell has been the subject of extensive academic interest, but the pathways involved in the process has not been fully elucidated. The mechanisms of cell-penetrating peptide entry into cells identified so far are direct translocation and endocytosis (Guidotti et al., 2017). Cell-penetrating peptide-drug couplers play an important role in the delivery of a wide range of drugs.

#### 3.1.2 Polymer drug delivery systems

Polymers play an important role in drug delivery due to their numerous types, unique three-dimensional structure, high drug loading capacity, and stable physicochemical properties. Polymers have evolved over the past few decades and have appeared in various forms such as hydrogels, polymeric films, and polymeric micelles. Polymers can be categorized into natural and synthetic polymers according to their origin. Natural polymers such as DNA, RNA, and peptides are widely used in bioengineering and drug delivery due to their unique properties (e.g., encodable, biodegradable). With the development of technology, advances in polymer modification techniques and the emergence of 3D printing technology, synthetic polymer carriers are becoming more versatile and powerful.

##### 3.1.2.1 Hydrogels

Hydrogels are highly porous crosslinked three-dimensional hydrophilic polymer networks of drug carriers that can swell by absorbing large amounts of water (Jindal et al., 2022). The presence of hydrophilic groups such as -CONH_2,_ CONH-, -OH, -COOH, -SO_3_H, *etc.*, is the main reason why hydrogels can absorb water and swell. In addition to their shared properties, hydrogels have individualized advantages based on their specific material. For example, chitosan and chitin have antimicrobial properties in their own right. So the antimicrobial capacity of gels based on them is further enhanced (Li et al., 2018). Guanine phosphate can stimulate innate immunity by binding to Toll-like receptor 9, and recognition of DNA hydrogels containing unmethylated cytosine-phosphate-guanine dinucleotides further enhances organismal immunity (Nishikawa et al., 2014). Biomaterials such as DNA and proteins, because of their encodable properties and similarity to the organism’s microenvironment, can cause some programmed changes in the gel, such as the spontaneous formation of artificial protein scaffolds (Sun et al., 2014). In addition, hydrogel materials can be further enhanced by adding functional groups or incorporating some functional materials to further enhance certain properties. Zhou et al. used a biomimetic mineral plastic of calcium carbonate and polyacrylic acid to give the gel optical properties (Zhou et al., 2020). Haraguchi et al. interwoven polymer and clay into a network to create a hydrogel that can repair itself after damage (Haraguchi et al., 2011). The incorporation of high aspect ratio nanoparticles into suitable gel materials can produce shear-thinning hydrogels, and they can protect cells from high shear forces, leading to a wide range of applications in cartilage tissue regeneration and cell delivery for 3D bioprinting (Thakur et al., 2016).

##### 3.1.2.2 Microsponges

Microsponges are a new type of drug delivery vehicle of porous polymer microspheres developed in recent years, usually consisting of crosslinked polymers with adsorbent capacity and containing a very large number of porous microspheres ranging from 5 to 300 μm inside, which can adsorb and encapsulate a large number of bioactive composition (Rahman et al., 2022; Tiwari et al., 2022). The microsponges ensure that the drug is localized on the skin surface and within the epidermis and does not diffuse in large quantities to non-diseased areas, preventing toxic effects caused by excessive accumulation of bioactive composition in the epidermis and dermis (Grabow and Jaeger, 2012). Green et al. used bionic microsponges to generate aggregates of inorganic particles that can provide surfaces and structures for cell attachment, organization and promote matrix synthesis (Green et al., 2020).

##### 3.1.2.3 Polymeric films

Polymer films, which are films made from polymer fibers, have great potential as topical drug carriers. Electrospinning is the fabrication of organized filaments of polymer nanofiber solutions under strong electric field forces, and the resulting fibers have finer diameters (0.1–100 nm) and larger surface areas than those obtained by conventional spinning methods (Bhardwaj and Kundu, 2010). The throughput of nanofibers has become a serious bottleneck limiting their application (Bhardwaj and Kundu, 2010). 3D printing is the latest technology for manufacturing polymeric films. 3D printing is a technology that uses virtual computer-aided design models to create physical objects by depositing successive layers (Economidou et al., 2018). By 3D printing, it is possible to create polymer films with different porosity, mechanical properties and drug loading to meet the needs of topical drug delivery in different situations. The need for materials with low viscosity, droplet size, lack of precision in positioning, high cost and long production time are factors that limit the widespread use of 3D printing technology for polymer films (Riccio et al., 2022).

#### 3.1.3 Ionic liquids

Ionic liquids are a new type of carrier, and everyone does not define it exactly the same way. Lei et al. considered ionic liquids to be compounds composed entirely of ions with melting points below 100°C (Lei et al., 2017). Gomes et al. considered ionic liquids as salts formed from organic cations and organic or inorganic anions (Gomes et al., 2021). Unlike conventional salts, it has unique physical and chemical properties. Although the definitions of ionic liquids are not identical, there is no doubt that ionic liquids are liquid salts at ambient temperature. Based on their chemical structure and properties, ionic liquids can be categorized into three groups: water- and air-sensitive ionic liquids, water- and air-insensitive ionic liquids, and biodegradable ionic liquids (Egorova et al., 2017). There are three applications of ionic liquids in increasing transdermal absorption of drugs: 1) Ionic liquids can be used as skin penetration enhancers for pharmaceutical formulations or in combination with other drug carriers. For example, the combination of ionic liquids and gels to form ionic liquid gels has been shown to be very effective in the treatment of infections and haemostasis (Luo et al., 2022; Gao et al., 2023). The mechanism by which ionic liquids promote transdermal absorption of drugs is not well understood, but may be negatively correlated with ionic interactions (Tanner et al., 2019). 2) The ionic liquid binds to the drug to form a drug-ionic liquid coupling. The essence of this approach is to convert the drug into a liquid salt so that the drug is characterized by the high stability and solubility of a liquid salt. Moshikur et al. combined 12 hydrophilic drugs with oil-soluble ionic liquids, which significantly increased the transdermal absorption of the drugs and significantly increased the elimination half-life and plasma concentration (Md Moshikur et al., 2022). 3) The ionic liquids are constructed by physical or chemical cross-linking to form new drug delivery systems poly-ionic liquids, such as poly-ionic liquid hydrogels.

#### 3.1.4 Deep eutectic solvents

Deep eutectic solvents are novel liquids made by mixing high melting point salts and molecular hydrogen bond donors, which can significantly change the melting point, solubility and biostability of drugs (McDonald et al., 2018). Deep eutectic solvents are usually composed of two or three inexpensive and safe components, which are capable of joining with each other through hydrogen bonding interactions to form low eutectic mixtures, and deep eutectic solvents with a high melting point are also chosen for some thermally unstable drugs to enhance the thermal stability of the drug (Zhang et al., 2012; Kumar and Nanda, 2018). However, how deep eutectic solvents promote transdermal drug absorption has been inconclusive. Boscariol et al. found that choline geranate deep eutectic solvent may have contributed to the penetration of bioactive molecules through the openings by sliding around the fatty compounds that make up the interstitial space between keratinocytes and by creating small transient openings in the cell membrane (Boscariol et al., 2021). However, the resulting small transient openings in the cell membrane did not cause damage to the skin tissue structure. In addition to this deep eutectic solvents can be involved in the stimulation of important components of the response element for precise drug delivery (Shi et al., 2022). Perhaps 1 day we will be able to use deep eutectic solvents to better achieve precise localization and dose control of transdermal drug absorption.

#### 3.1.5 Liquid crystals

Liquid crystals are a state of matter between a solid and a liquid, having both fluidity like a liquid and an orderly arrangement of crystals (Dierking and Al-Zagana, 2017). In 1957, Brown and Shaw described the physical properties of liquid crystals in their book (Mitov, 2017). Liquid crystals can be classified into a number of categories based on the conditions under which the liquid crystal state is maintained. The most commonly used classification of liquid crystals is the division of liquid crystals into thermotropic liquid crystals, which maintain the liquid crystal state within a certain temperature range; and lyotropic liquid crystals, which maintain the liquid crystal phase state under specific concentration conditions. The difficulty in realizing thermotropic liquid crystals at ambient temperature is the reason why almost no thermotropic liquid crystals are used in drug delivery systems. However, it has recently been found that thermotropic liquid crystals can alter the spontaneous water flux at the liquid crystal phase and interface by changing the type and concentration of the added electrolyte, further reorienting the liquid crystals at the aqueous interface and thus releasing the substances stored in the liquid crystals (Ramezani-Dakhel et al., 2017). In this way, thermotropic liquid crystals have the possibility and potential to become drug delivery systems that can precisely control drug release. The liquid crystallinity of lyotropic liquid crystal is a function of concentration and is mostly composed of amphiphilic molecules, the most common types being cubic, hexagonal and layered intermediate phases (Chavda et al., 2022). Many lyotropic liquid crystals have a lipid structure with similarities to the stratum corneum and strong bioadhesive properties, giving them an important place in transdermal drug delivery systems (Lotfy et al., 2022).

#### 3.1.6 Metal-organic frameworks

Metal-organic frameworks are a class of highly ordered crystalline porous coordination polymers formed by the coordination of metal (transition metal or lanthanide metal) ions and organic ligands (carboxylates, azides, and phosphonates) that adsorb functional molecules on external surfaces or in open channels and trap these molecules in the backbone (Sun et al., 2020; Cheng et al., 2022). Metal-organic frameworks are produced by hot-melt, microwave-assisted synthesis, ultrasonic-assisted synthesis, *etc.* The metal-organic frameworks synthesised by different methods affect their crystal structure, size, porosity and other properties. Metal-organic frameworks can be used in conjunction with the formation of nanomaterials containing the active ingredient of a drug or with stimulus-responsive elements for the fine delivery of localised drugs (Zhao et al., 2019; Pan et al., 2022).

#### 3.1.7 Bacteria and bacterial derivatives

Bacteria and bacterial derivatives, including bacterial ghosts and extracellular vesicles, are very promising bio-nanomaterials. Drug delivery platforms of bacteria and their derivatives retain some of the autonomous and dynamic functions of bacteria, such as colonization and targeting of human tissues, tissue penetration ability and enhanced activation of the body’s immune response (Chen et al., 2021). Compared with chemically synthesized drug carriers (nanoparticles and liposomes, *etc.*), drug carriers composed of natural biomaterials have both active and passive targeting properties (Cao and Liu, 2020). Endowed by means of engineering, bacteria and their derivatives can also be endowed with additional functions (Li et al., 2021).

##### 3.1.7.1 Bacteria

Bacteria are closely associated with human health and disease development, playing a key role in preventing and maintaining atherosclerosis, fighting skin cancer, promoting skin wound healing and hair follicle rejuvenation, and neurological disorders including depression (Gilbert et al., 2013; Li et al., 2016; Naktsuji et al., 2018; Wang et al., 2021; Qiao et al., 2022). There are two common bacterial drug carriers (Li et al., 2021). One is the integration of disease-treating genes into the bacterial chromosome by means such as CRISPR-Cas9, which is developed into a living biopharmaceutical, allowing the drug to be continuously produced as the bacteria metabolize. The other is to load the drug into the living bacterial body to release the drug with bacterial lysis. Vishnu et al. studied and developed a non-pathogenic, therapeutic delivery system for *Salmonella* strains that can efficiently deliver bioactive proteins into cells by utilizing genetic circuits to control protein synthesis, invasion into the cell and release of protein drugs. And it can deliver drugs directly to cancer cells specifically without affecting healthy cells (Raman et al., 2021). The combination of the bacterial carrier effect with its own effects such as enhancing the immune response will certainly lead to better therapeutic results. In the future, the bacterial drug delivery system may be able to deliver drugs as precisely as an intelligent robot.

##### 3.1.7.2 Bacterial ghosts

Bacterial ghosts are produced by the release of bacterial cytoplasmic contents through channels in the cell envelope, mainly from Gram-negative bacteria. The high number of cell wall layers, strong mechanical properties, the presence of more toxic exotoxins and the absence of a relatively simple protein secretion mechanism and high transporter capacity may be the reasons why Gram-positive bacteria are generally not used as delivery vectors but as vaccines (Hjelm et al., 2015; Wu et al., 2017). There are two methods of creating bacterial ghosts, the chemical-based “sponge-like” method and the genetically-engineered method, which is prepared by cleavage of gene E. The method is based on the use of chemical reagents. The “sponge-like” method refers to the use of chemicals to create pores through the bacterial cell wall, followed by centrifugation to remove the cell contents (Chen et al., 2021). The genetic engineering method utilizes the cleavage protein E produced by the cleavage gene E to cause the formation of transmembrane tunnels connecting the inner and outer membranes near the bacterial division site, flowing out of the cytoplasm and producing bacterial ghosts with surface structures identical to those on the surface of living cells (Ma et al., 2021). Beyond that, some bacterial ghosts have the ability to target infected macrophages (Xie et al., 2020). Combining bacterial ghosts with micro needling or subcutaneous injections would have great potential for application in bacterial infections of the skin or deep tissues.

##### 3.1.7.3 Bacterial extracellular vesicles

Bacterial extracellular vesicles are spherical membrane particles with diameters of 20–400 nm secreted by commensal or pathogenic bacteria, which can be categorized into cytoplasmic membrane vesicles and outer membrane vesicles according to the type of bacteria secreted (Liu et al., 2022). Cytoplasmic membrane vesicles are derived from the cytoplasmic membrane of Gram-positive bacteria carrying material from the cytoplasm. Outer membrane vesicles are derived from the outer membrane of Gram-negative bacteria carrying material from periplasmic and cytoplasmic components (Toyofuku et al., 2019). Bacterial extracellular vesicles are the language of communication between bacteria and hosts, and can be used to regulate a variety of biological functions including biofilm formation, alteration of small intestinal epithelial permeability (Fizanne et al., 2023), increase in host angiogenesis and osteogenesis (Chen et al., 2022), inhibition of host viral infections and host collaboration in viral clearance (Ñahui Palomino et al., 2019; Bhar et al., 2022), induction of host resistance to other bacteria (Zhou et al., 2023), and host immunomodulation (Xie et al., 2022). Bacterial extracellular vesicles can release the contents of bacterial extracellular vesicles into host cells through three ways: endocytosis, internalization of bacterial extracellular vesicles through lipid rafts, and direct membrane fusion (Ñahui Palomino et al., 2021). Endocytosis is considered to be the main route of entry of bacterial extracellular vesicles into eukaryotic cells.

#### 3.1.8 Cells and cell derivatives

Cells and cell derivatives, including cell membranes and cell vesicles, are drug delivery systems that modify drugs to the surface of cell membranes, encase them inside the living cell membranes, or integrate them onto cellular nucleic acids. When modified cells are delivered into a patient, such as by integrating gene fragments onto nucleic acids or wrapping around the inside of cells, they can become a kind of “living drug”, constantly producing drugs as cells metabolize or releasing a certain number of drugs as special events occur. Differences in the composition of their membranes, types of receptors, *etc.*, of cells and cell derivatives of different origins have the ability of active/passive targeting to specific tissues. Some cells and cell derivatives also have the ability to activate the immune system, which can assist drugs to better treat diseases. Cells and cell derivatives have a wide scope for development in precision therapy and as controlled targeted drug carriers.

##### 3.1.8.1 Cells

A cellular drug carrier is one that utilizes an intact cell or a denucleated cell as a carrier. In contrast to being intact, denuded cells not only do not proliferate or permanently engraft in the host, they also retain organelles to produce energy and proteins. Desmoplastic cells adhere to target cells or tissues through integrin-regulated adhesion and their functional properties are highly similar to those of extracellular vesicles (Wang et al., 2022). Red blood cells are unique in that they are whole cells and do not possess a nucleus of their own, allowing them to be transported to tissues through the bloodstream. Mesenchymal stem cell, with their intrinsic disease-targeting and paracrine capabilities, have gained a great deal of attention as therapeutic vectors. The short lifespan of neutrophils means that drugs can enter the bone marrow as senescent neutrophils return to the bone marrow. In addition, cells such as hematopoietic stem cells, platelets, natural killer cells, macrophages, dendritic cells, T-lymphocytes and tumor cells have also been used as drug delivery vehicles. The combination of these cells with topical implantation techniques or microneedling could have a promising future in the topical treatment of diseases of the skin and subcutaneous tissues.

##### 3.1.8.2 Extracellular vesicles

Extracellular vesicles are nanoparticles encapsulated by lipid membranes secreted by cells, and are widely distributed in various body fluids, tissue fluids and cell culture supernatants (Hallal et al., 2022). Extracellular vesicles are both a means of disposing of harmful or unwanted intracellular components and are capable of long-range intercellular communication *in vivo* surface proteins, encapsulated cargo molecules (e.g., biologically bioactive molecules such as proteins, lipids, nucleic acids and sugars) (Shao et al., 2018; Buzas, 2023). Depending on the source, particle size, structure and function, extracellular vesicles can be categorized into various types such as exosomes, microvesicles, apoptotic bodies and oncosomes. The first extracellular vesicles identified were those loaded with transferrin receptors, which were excreted by reticulocytes via exosomes (Harding et al., 1984). With the exploration of extracellular vesicles, the therapeutic potential of extracellular vesicles in various diseases continues to be discovered.

##### 3.1.8.3 Cell membrane

Cell membrane is a drug carrier in which the cell membrane of a certain cell is wrapped around the outer layer of the drug. Erythrocytes, leukocytes, platelets, cancer cells, macrophages, mesenchymal stromal cells, and exosomes have been used as cell membrane drug carriers. They closely resemble cell membrane components *in vivo* and carry a large number of surface molecules, possessing similar functions to cells and exosomes. Different types of cell membranes also possess their individualized functions. For example, drugs encapsulated in natural erythrocyte membranes inhibit drug uptake by the reticuloendothelial system (Rao et al., 2016). The membranes of macrophages, leukocytes and monocytes have a high affinity for inflamed and tumor-bearing regions, easily pass biological barriers, and can migrate transendothelially (Narain et al., 2017). The cell membranes of platelets have subendothelial adhesion properties as well as the ability to interact with pathogens (Kunde and Wairkar, 2021). Human keratinocyte membranes can achieve precise targeted release of drugs in the skin, which has a broad application prospect in dermatological treatment and functional cosmetic delivery (Jing et al., 2021).

#### 3.1.9 Inorganic drug carriers

Drug carriers made from organic materials can easily dissolve and encapsulate poorly water-soluble drugs into their hydrophobic cores, but are also physicochemically and chemically unstable, making them susceptible to accidental drug leakage. Drug carriers made from inorganic materials such as mesoporous silica nanoparticles, carbon nanomaterials and gold nanoparticles are highly physicochemically and biochemically stable, less expensive to manufacture, and can provide sufficient attachment sites for a variety of organic components. Many inorganic drug carriers can also be prepared in a variety of sizes, structures and geometries. Compared to some highly biocompatible organic carriers, inorganic carriers are less able to cross the skin barrier, but the high drug loading capacity can be used as a strategy to control the retention and release of therapeutic candidates.

##### 3.1.9.1 Silicon dioxide

Silicon dioxide drug carriers are drug carriers made of silicon dioxide, first synthesised in the 1990s, and are available in spherical, rod and flake shapes (Vallet-Regí et al., 2022). A type of silica porous microspheres with pore sizes ranging from 2 to 50 nm has occupied an important position in the family of silica drug carriers since its discovery, which is mesoporous silica (Vallet-Regí et al., 2022). In addition to this, many researchers have structurally modified mesoporous silica to give mesoporous silica a richer and more personalized advantage. For example, Xu et al. utilized a chiral amide gel-directed synthesis method that could give mesoporous silica significant chiral activity (Xu et al., 2023). Shiekh et al. modified mesoporous silica with hexamethylsilazane, which avoided agglomeration and was more conducive to targeted delivery (Shiekh et al., 2022). Difficulty in entering the bloodstream through the human stratum corneum even with the addition of chemical penetrating agents is the main obstacle that restricts the use of mesoporous silica drug carriers for some topical drugs. However, Zhao et al. utilized the drag effect of low eutectic solvents to enable mesoporous silica drug carriers to effectively penetrate the entire skin (Zhao et al., 2022).

##### 3.1.9.2 Calcium carbonate

Calcium carbonate is one of the most widespread minerals found in nature and is also widely found in living organisms as a structural support. Calcium carbonate exists in various forms, such as amorphous and crystalline. There are six known crystalline forms of calcium carbonate, namely, calcite, aragonite, spherulite, calcium carbonate monohydrate, calcium carbonate hexahydrate, and calcium carbonate hemihydrate. The Ca^2+-^ CO_3_^2-^ reaction system is the most commonly used way to synthesize calcium carbonate. Calcium carbonate with different sizes, morphologies and crystalline forms can be obtained by changing the reaction conditions (Niu et al., 2022). Surface modification is the main method to expand the application of calcium carbonate in drug carriers. Calcium carbonate was further engineered into porous, hollow or core-shell organic-inorganic nanocomposites by organic modification or inorganic modification, followed by template-induced biomineralization and layer-by-layer assembly of the resulting CaCO_3_ (Niu et al., 2022). Dong et al. utilized hollow calcium carbonate-polydopamine composite nanomaterials to fabricate a photosensitizer-loaded drug carrier that could significantly reduce phototoxicity and thus effectively minimize skin damage during *in vivo* antitumor photodynamic therapy (Dong et al., 2018).

### 3.2 Drug-smart response element

Drug-smart response elements are elements that can be targeted for delivery to a focal site after receiving a specific external or internal stimulus. In contrast to conventional drugs, stimuli-responsive elements can use a variety of principles to respond to subtle changes in the body’s environment, thereby delivering the drug precisely to the target site, which can reduce unwanted toxic side effects of the drug at non-target sites without increasing the concentration of the drug (Table 4).

**TABLE 4:** Classification, advantages, disadvantages and applications of drug-smart response elements.

Drug-smart response element	Classification	Advantages	Disadvantages	Applications	Ref
Ligand-mediated of Drug-smart Response Element	Endogenous	1. Highly specific targeting ability of antibodies when used as ligands	1. Weak penetration ability when antibody is used as a ligand	Ligand-mediated drug-intelligent response elements are widely used in tumour therapy, facilitating transdermal absorption of drugs and targeted drug delivery to abnormal regions	Yang et al. (2012), Wang et al. (2020), Nie et al. (2021), Fu et al. (2022), Katila et al. (2022)
		2. Small molecule compounds can enhance the ability of drug penetration barrier when used as ligands	2. Low targeting ability when small molecule compounds are used as ligands		
pH-Responsive Drug Smart Response Element	Endogenous	Many abnormal physiological activities may trigger changes in local microenvironmental pH	Multiple pH Alterations in Complex Diseases pH-responsive drug smart response elements are less selective	pH-responsive drug smart response elements can be applied to anticancer and antibacterial drugs	Stanton et al. (2017), Yang et al. (2018), Zhou et al. (2020), Li et al. (2022)
Redox-responsive Drug Smart Response Element	Endogenous	In pathological states, the body’s oxidising or reducing substances are then altered accordingly, which in turn disrupts the redox balance homeostasis	Redox-responsive drug smart response elements are costly and unstable *in vivo*, with the risk of inducing immunogenic responses, frequent drug release lags and delayed drug action	Redox-responsive drug smart response elements may be used as oncology therapeutic agents or as adjuvant oncology therapeutic agents	An et al. (2019), Herold et al. (2020), Lu et al. (2021), Wang et al. (2021), Li et al. (2022)
Enzyme-responsive Drug Smart Response Element	Endogenous	Enzyme-responsive drug smart response elements with mild reaction conditions, high catalytic efficiency and specific substrate selectivity	Most of the Enzyme-responsive drug smart responses have stringent substrate requirements and are difficult to design	Enzyme-responsive drug smart response elements can be applied to oncology drugs and antimicrobials	Do et al. (2022), Wu et al. (2023)
Magnetically Responsive Drug Smart Response Element	Exogenous	Magnetic-responsive drug-smart response elements are non-invasive, easy to handle and well biocompatible	Magnetic-responsive drug-smart response elements require equipment that generates the required magnetic field	Magnetically Responsive Drugs Smart Response Elements can be used on antimicrobial drugs or synergistically with the localised thermal effects produced by magnetothermal therapy to kill diseased cells	Stanton et al. (2017), Song et al. (2020), Du et al. (2022), Lee et al. (2023)

#### 3.2.1 Ligand-mediated of drug-smart response element

Chemical modification has been an important way to improve the performance of drugs or carriers. Ligand-drug couplings are one such chemical modification method that has received widespread attention, first appearing in the 1870s (Chau et al., 2019). Small molecules have since been used as ligands, but antibodies as ligands have been the most dominant research direction. Currently, antibody couplings are developing rapidly. In addition to enhancing the targeting ability of drugs, antibody couplers can also activate the body’s immune response and regulate cell behaviour (Qian et al., 2021; Liu et al., 2023). The choice of antibodies is not limited to proteins with direct potency similar to those in the human body, but also includes bacterial surface proteins with indirect potency (Lahav-Mankovski et al., 2020). Some researchers have found that further modification of bacterial antibodies can give bacteria programmable properties to more finely regulate the organism by inducing cellular functions in a manner similar to cell surface protein responses. However, antibody couplings have a poor ability to penetrate barriers, and intravenous injection is the predominant route of administration. The application of microneedling and embedding technologies may be an important step in further expanding the application of ligand-mediated drug smart response elements.

#### 3.2.2 pH-responsive drug smart response element

pH-Responsive drug smart response element is a drug delivery system that combines a pH-responsive structure, such as a 3+ carboxylic acid ligand, through surface adsorption, surface modification and mixing (Guillen et al., 2022; Tian et al., 2022). The most commonly used design is the modification of the carboxyl group of a protein with a stilbene analogue, which binds to the biologically active ingredient of a target drug having p-dimethylaminobenzaldehyde or to a drug carrier (Herold et al., 2020). pH-responsive drug smart response elements allow for the slow, sustained release of drugs from the smart response element at varying rates depending on the acidification conditions of the human environment, and have great potential for the long-term sustained release of drugs. *In vivo* pH-responsive drug smart response elements have major limitations. However, pH-responsive drug smart response elements have great applications in skin bacterial infections and trauma (Xie et al., 2023).

#### 3.2.3 Redox-responsive drug smart response element

Redox-responsive drug-smart response elements typically contain disulfide and diselenide bonds that load the active pharmaceutical ingredient in a covalent or non-covalent manner and release the active pharmaceutical ingredient in response to oxidative stimulation (Li et al., 2022). The most commonly recognized substrates are reactive oxygen species and glutathione. The very slow kinetics of the classical “thiol-disulfide bond exchange reaction” is a major drawback of redox-responsive drug-smart response elements. Relief through the addition of core gating molecules can improve the accuracy of drug release and enable more precise disease targeting (Li et al., 2022). Because bacteria contain high concentrations of glutathione, the redox-responsive drug smart response element is likely to clear the bacteria (Wu et al., 2023). The skin is more prone to artificially modulate drugs, and redox-responsive drug-smart response elements have great potential for skin or mucosal antimicrobials.

#### 3.2.4 Enzyme-responsive drug smart response element

The design principle of enzyme-responsive drug smart response elements is to utilize specific interactions between enzymes and substrates to prepare drug-smart response elements that can be specifically degraded by specific enzymes (Zhou et al., 2022). Precise localisation of enzyme-drug smart response elements could control the drug release dose according to disease severity and greatly reduce cytotoxicity (Zuo et al., 2020; Chen et al., 2021). In addition to this, enzyme-drug smart response elements have some personalized advantages. Self-assembled peptide thioesters consisting of aminoethyl thioesters as substrates for thioesterases can act on the Golgi apparatus of target cells in a timely and efficient manner via an enzymatic reaction, leading to cell death through a variety of pathways (Tan et al., 2022). Enzyme-responsive drug smart response elements can deliver live drugs such as cells and bacteria. Yang et al. solved the problems of low cell survival and severe immune rejection by utilizing matrix metalloproteinase-7-responsive nanoshells to encapsulate HeLa cells and human mesenchymal stem cells (Yang et al., 2019).

#### 3.2.5 Magnetically responsive drug smart response element

Magnetic nanoparticles are 10–100 nanoparticles represented by iron, cobalt, nickel or metal oxides with inherent magnetic properties are the main source of magnetically responsive targeted drug delivery systems (Shi et al., 2022). Superparamagnetic magnetic hematite (γ-Fe2O3) or magnetite (Fe_3_O_4_) are commonly used magnetic nanoparticles in magnetically responsive drug smart response elements. Micro-robotics is the most ideal development direction for magnetically responsive drug smart response elements. An ideal micro-robotic drug smart response element should fulfill four core requirements: 1) high loading capacity, 2) protection of the drug from the external environment, 3) controllable propulsion mechanism, and 4) on-demand drug release (Song et al., 2022). However, achieving the above conditions using magnetically responsive elements is a long way from controlling the micro-robot to deliver the drug to the specified location.

### 3.3 Factors affecting transdermal drug absorption

Permeation of drugs through biological or synthetic membranes occurs through three main modes of transport: passive, active or facile. Transdermal absorption in humans or animals is the passive diffusion of drugs from carriers or excipients on skin surface tissues to reach the systemic circulation (Schaefer et al., 2013). The physiologic structures involved in the transdermal absorption of topical medications are the stratum corneum, hair follicles, sebaceous glands, and sweat duct orifices. The pathways of topical drug penetration through the stratum corneum can be categorized into three: 1) Intercellular pathway: chemicals bypass the keratinocytes and penetrate into the subcutis through the intercellular matrix that is continuously distributed between the keratinocytes (Barbero and Frasch, 2006). This is the main route for bioactive composition in drugs with very small molecular weights to enter the skin. The bioactive components of drugs that enter the skin via the intercellular route are of low molecular weight and have a certain degree of lipid- and water-solubility (David, 2014). 2) Transcellular entry: the chemical enters the target site either directly through keratinocytes and mesenchyme or through efflux and uptake transporters using transporters (Haque and Talukder, 2018; Bajza et al., 2020). The bioactive composition with smaller molecular weights directly pass through the cell membrane, while the components with larger molecular weights interact with transport proteins and pass through the cell membrane through efflux and uptake transporters (Idson, 1975). 3) The transdermal route by which chemicals enter the dermis directly through skin appendages such as hair follicles, sebaceous glands and sweat duct orifices is also known as the bypass route. In most cases, drug bioactive composition with large molecular weights or peculiar structures have difficulty in passing through the thicker lipid-rich stratum corneum and enter the skin mainly via the bypass route (Stoughton, 1965). The total area of the skin appendages is relatively small, but it has a significant impact on the skin permeability of drug bioactive composition (Marwah et al., 2016). The bypass pathway is an important reason why topical drugs resemble slow-release drugs. The storage of drugs in the skin appendages and the extremely thin stratum corneum of the skin appendages allow large molecular weight drug bioactive composition to cross the skin slowly at a constant rate for a certain period of time to reach the target site (Scheuplein and Blank, 1971; Ishii et al., 2010).

The nature of drug absorption on the skin is a passive diffusion process from the outer part of the skin, where the concentration is high, to the inner regions of the skin, where the concentration is low. In principle, all three pathways follow Fick’s law of diffusion, which states that the flux or motion of a molecule diffusing across a membrane is proportional to the difference in concentration of that molecule on either side of the membrane (Idson, 1975). The formula based on Fick’s first law of diffusion indicates that the maximum flux of a drug is proportional to the difference in skin concentration and inversely proportional to the thickness of the stratum corneum. Fick’s second law of diffusion suggests that the relationship between diffusion distance and the duration until an isopore (an area of the same drug concentration) is reached is not linear, but increases disproportionately with increasing diffusion distance (Wohlrab and Eichner, 2023). However, the efficacy of some drugs decreases significantly with increasing drug concentration (Hemmati et al., 2011), indicating that in addition, there are other important factors that similarly influence drug penetration and absorption. The integrity and properties of the membrane (skin) as the main body for the penetration of the bioactive composition of the drug have a great influence on the penetration of the drug (Gattu and Maibach, 2011). Lipid-water partition coefficients, solubility, melting point, molecular size and shape are widely recognized as important physicochemical factors that influence skin penetration and absorption of drug bioactive composition by their own properties. The strength of the effect of different factors on the penetration of bioactive composition of drugs is closely related to the structural composition of the skin and the prevailing skin condition (Lien and Tong, 1973; Michaels et al., 1975; Roberts et al., 1977). Magnusson et al. found that epidermal permeability coefficient and solute octanol-water partition coefficient were the key factors in the permeation efficiency of pharmaceutical bioactive composition (Magnusson et al., 2004). Molecular weight is an important factor in determining the maximum permeation flux of a pharmaceutical bioactive compound. The permeation of pharmaceutical bioactive compounds is also related to the specific surface area of the particles, the diffusion coefficient of the solute, the thickness of the boundary layer and the solubility of the solute (Sandri et al., 2014). The rate of permeation of a solute from an aqueous solution depends on the efficiency of partitioning and diffusion. The efficiency of solute partitioning in solution is related to the octanol-water partition coefficient. The efficiency of solute partitioning in solution is related to solute size and hydrogen bonding (Zhang et al., 2009). Passive diffusion of drugs through the skin is influenced by the physicochemical properties of the drug bioactive composition and by individualized differences in the structure and physiological state of the skin. However, not all drug bioactive composition penetrates at a higher rate in damaged skin than in normal skin. For example, nicotine transmits at a higher rate through normal healthy skin than through skin previously damaged by corrosive or destructive chemicals or physical agents (e.g., heat and cold) (Macht, 1938). Hydration not only plays an important role in skin barrier function, but also plays a major role in promoting the absorption of drug bioactive composition. Hydration of the stratum corneum can lead to profound changes in skin barrier properties. In its normal state, the stratum corneum contains 15%–20% water, and stratum corneum water can increase to about 400% after over-soaking (Dawber, 1980). Upon absorption by keratinocytes, water alters the position and stability of the disulfide bonds in keratin peptides, causing changes in the spatial orientation of the protein (Walters, 2002). The change in the spatial orientation of the proteins gives more space to the water molecules, allowing the water content of the keratinocytes to increase further. When the keratinocytes themselves swell, the denseness of the structure decreases, the cellular gap between the keratinocytes widens, and the permeability of the skin increases. The hydration of the skin can change the physicochemical properties of the bioactive composition of the drug and increase the diffusion coefficient of the bioactive composition of the drug. As the water content of the keratinocytes increases, the bioactive composition of the drug are more likely to combine with water to form hydrated molecules (Scheuplein et al., 1969; Wiedmann, 1988). In addition to this, pH and temperature also affect the transdermal absorption of drugs. pH is not absolute in its effect on the transdermal absorption of the bioactive composition of a drug. For example, the transdermal absorption dose of indomethacin increases as the pH of the skin surface decreases, a pattern that is not necessarily true for other pharmaceutical bioactive composition (Chiang et al., 1991). Alteration of pH affects the ionization of drug bioactive composition may be the main reason why pH affects the transdermal absorption of drug bioactive composition. Unlike pH, an increase in temperature generally increases the transdermal absorption of drug bioactive composition. Temperature increases skin blood flow at the site of heat application mainly by promoting skin vasodilation, which significantly increases the rate of transdermal absorption of drugs and the dose of transdermal absorption (Barkve et al., 1986; Hao et al., 2016).

## 4 Individualized drug use that affects the effectiveness of individualized dosing

The effect of the same drug on different individuals can vary greatly. The main reasons for this difference are family heredity, disease, age, weight and the concurrent use of other drugs. Personalized medicine refers to the formulation of a more reasonable treatment plan with full consideration of each patient’s individual situation. Thus, improving the efficacy of drugs, reducing the side effects of drugs in a more economical way achieve better therapeutic effect.

### 4.1 Pharmacogenomics affecting the effectiveness of individualized dosing

As everyone knows genetic sequence varies from person to person by only one in a thousand. Deletion, insertion, duplication and inversion are the four genetic structural variants that are always present. Metabolic enzyme, transporter, receptor and other genes encoding metabolic enzyme regulate the absorption, distribution, metabolism and excretion of substances in the human body. This makes the same concentration of the drug have different therapeutic effects and adverse effects on people with different genotypes. Large studies have shown that adverse drug reactions occurring in hospitals are one of the leading causes of death among American hospital patients (Roden et al., 2019). 97%–98% have at least one functional drug-related gene variant (Schärfe et al., 2017). These studies have shown that exploring human drug-related genes is particularly important for individualising medication and improving drug efficacy, hence the emergence of pharmacogenomics. Metoprolol is an important drug in the treatment of various cardiovascular diseases, thyroid crisis and localised choroidal haemangiomas (Zamir et al., 2022). The metabolism of the polymorphic enzyme CYP2D6 is the main reason that affects the metabolic rate of Metoprolol *in vivo*. In one study, the genotypes of patients were 30% extensive metabolizers, 55% intermediate metabolizers, and 13% weak metabolizers (Anstensrud et al., 2020). If the same drug dose is used in people with the CYP2D6 weak metabolism genotype, the risk of adverse reactions during Metoprolol treatment is 5 times higher than in people with the non-weak metabolism genotype (Bijl et al., 2009). Adjusting Metoprolol dosage according to genotype significantly reduces the probability and severity of adverse reactions in patients. More and more genotypic and phenotypic information related to the pharmacogenome is being discovered and many very useful databases have been established. As of April 2021, PharmGKB contains 715 drugs 1761 genes, 227 diseases, 165 clinical guidelines, and 784 drug labels (Gong et al., 2021). In PharmGKB, we have access to genotypic and phenotypic information related to the pharmacogenome. It includes drug dosing guidelines, drug labelling annotations, clinical and variant annotations, summary of drug-centred pathways, pharmacodynamic genes and the relationship between genes, drugs and disease. Approximately 4/5 patients may carry a variant that is a target for commonly prescribed drugs and may also have a functional impact, a variant that may alter drug efficacy (Schärfe et al., 2017). The correct use of gene sequencing tools and pharmacogenomics-related databases can effectively guide patients in the rational use of medication, improve drug effectiveness and reduce adverse drug reactions.

### 4.2 Microbiomes influencing the effectiveness of individualized dosing

The human organism contains hundreds of microorganisms with different biochemical functions that are relevant to human life. In recent decades, researchers have discovered that microorganisms also play a role in drug metabolism and efficacy. Zimmermann et al. analyzed 76 bacterial species, metabolizing 271 oral drugs *in vitro* and found that at least one strain was able to metabolize 2/3 of the drug (Zimmermann et al., 2019). Chemical modification of microorganisms is an important cause of altered drug efficacy. 5-aminosalicylic acid has a significant inhibitory effect on intestinal wall inflammation (Tavares Junior et al., 2022). With increased use, resistance develops in most patients. Anaerobic faecal culture experiments have shown that up to one-third of 5-aminosalicylic acid is metabolised by microorganisms to a form that lacks anti-inflammatory activity, N-acetyl-5-aminosalicylic acid (Dull et al., 1987). Mehta et al. identified 12 previously uncharacterized enzyme genes associated with 5-aminosalicylic acid inactivation and found that an increase in the number of these enzyme genes correlated with steroid use (Mehta et al., 2023). Metabolites of microorganisms can also affect the efficacy of drugs. Koh et al. found higher levels of imidazolepropionic acid, a metabolite of the gut flora, in patients who were not well treated with metformin (Koh et al., 2020). Further studies showed that imidazole propionate inhibited metformin-induced adenosine 5′-monophosphate-activated protein kinase activation via p38g and Akt, whereas pirfenidone blocked p38γ activation by imidazole propionate and restored the hypoglycaemic effect of metformin inhibited by imidazole propionate. Metabolism of drugs by microorganisms is the source of some adverse drug reactions. Clonazepam is an antiepilepsy and anxiolytic drug, and the breakdown product of this drug, vinylpyrimidine bromide, is severely toxic. Zimmermann et al. found that the serum level of bromoacetyluracil was five times higher in normal mice than in germ-free mice after the use of clonazepam (Zimmermann et al., 2019). The microbiome is relatively new and many of the current studies do not directly benefit patients, but there is no doubt that microbial metabolism has a huge impact on drug metabolism and efficacy. Moreover, the current microbiome research mainly focuses on the intestinal flora, and the research on skin microorganisms is relatively backward, which is not conducive to the research on the individualization of topical medications. Analyzing the reasons why drugs are metabolized by microorganisms can link individual differences in the microbiome to individual differences in drug metabolism, which can be targeted to accurately guide patients in the rational use of medication by restoring the efficacy of the drug or reducing the adverse effects of the drug.

### 4.3 Artificial intelligence modelling of the effectiveness of individualized dosing

Over the past decade, artificial intelligence (AI) has undergone a revolution that is bound to transform economics, society and science, solving many seemingly intractable problems (Richards et al., 2022). In the field of pharmaceuticals, AI has achieved excellent results in data processing, drug discovery (Schneider et al., 2020), image processing (Bera et al., 2021), and chemical structure analysis (Bojar and Lisacek, 2022). However, due to reasons such as medical datasets being difficult to access and the medical field being too large and complex. AI has not really made a real difference in the medical field. As it stands, assisting in disease identification and classification is one of the most promising aspects of AI. Accurate disease delineation and documentation can assist us in identifying and addressing more subtypes of disease further enabling individualized drug delivery. In this context, Acosta proposed a multimodal medical AI model that processes multiple types of information (Acosta et al., 2022). In this model, the AI model is able to make good use of data from both clinical and non-clinical databases, thus truly enabling AI to diagnose and treat diseases. Moor proposed another new paradigm of medical AI (Moor et al., 2023), known as the model of holistic medical AI. This model labelled data, flexibly interpreting data from different combinations of medical patterns and producing expressive outputs through self-supervision of large, diverse datasets. The models of omnipotent medical AI have the ability to customize queries to interact with the model compared to multimodal medical AI models, flexible combinations of different data modalities can be received and results output, allowing inference using undirected tasks. Multimodal medical AI models and all-round medical AI models require a diverse and specialized large database, which is very difficult and requires a very large financial investment. The processing of high-throughput data and the high accuracy requirements of models are also important issues that cannot be ignored in both types of modelling. AI is generally superior to individual physicians in data processing, and it can perform more complex information processing, find new subtypes of disease, and discover links between an individual’s characteristics and medication use, leading to better and more precise selection of medication types and dosages for an individual, and thus improving medication efficacy.

## 5 Conclusion and outlook

More than half of the world’s population uses at least one drug per day, and as the global burden of disease continues to grow, the demand for drugs will continue to increase (Baryakova et al., 2023). Patient compliance is an important factor in disease treatment. Statistically, poor adherence leads to 10% of hospitalizations, resulting in $100 - $300 billion in avoidable healthcare costs and causing approximately 125,000 U.S. patient deaths annually (Baryakova et al., 2023). Oral medication is the most common and highest patient compliance route of medication use. However, most oral medications are not specific and may cause side effects. Metabolism in the gastrointestinal tract and liver can further reduce the dose of bioactive ingredients reaching the target site. Topical administration is a promising and effective method of drug delivery. Topical medication is a highly compliant mode of administration, is not metabolised in the gastrointestinal tract and has a high specificity for skin tissues.

Dermatological diseases are the greatest advantage of topical medication. Topical drugs can quickly reach the target site of skin diseases, shorten the time for the drug to produce therapeutic effects, and avoid the adverse effects of drug hoarding in other tissues. The skin is a complex and sophisticated tissue composed of microorganisms that inhabit the surface layer, cells that make up the structure of the skin, immune cells and their metabolites that are involved in innate immunity, adaptive immunity, and that help to protect the organism against external aggressions, guaranteeing the organism’s ability to survive in a harsh and dry external environment. The effectiveness of topical treatments is influenced by a number of factors. The transport of the bioactive composition of a topical drug from the skin to the target site is a key step in the efficacy of topical drugs. Regardless of the target site of the disease, the bioactive composition of topical drugs needs to cross the stratum corneum barrier. Dermatological diseases in which the bioactive composition crosses the stratum corneum barrier and reaches the target site directly to take effect. In subcutaneous tissue diseases, the bioactive composition has to be further diffused and cleared by metabolism and dermal circulation to reach deeper tissues. In diseases of other tissues and organs, bioactive composition also needs to be transported through the bloodstream to reach the target site. Enabling drug penetration through the skin is a major concern for topical drug delivery. The state of the skin barrier, the physicochemical properties of the bioactive composition, the excipients and additives of the agent, the drug delivery system and external means of altering the efficiency of transdermal absorption, such as heat and iontophoresis, all affect the efficiency and dose of the bioactive composition in reaching the target site, and thus the effectiveness of the bioactive composition. Undeniably, the question of how to enhance the transdermal absorption of drugs into the skin is a very important one. The advent of drugs of enormous size, such as proteins, nucleic acids and bacteria, has led to a demand for methods to increase the transdermal absorption of drugs. However, enhancing transdermal absorption of drugs is not the first option to improve the effectiveness of all drugs. Drugs with small molecule compounds as the main bioactive composition are still the most commonly used topical drugs in the clinic. Small molecule actives usually have a very good ability to penetrate the skin. Excessive promotion of transdermal absorption of drugs containing small molecules of biologically active ingredients results in most of the biologically active ingredient entering the bloodstream, while less of the drug is available at the target site of the skin, thus reducing the efficacy of the treatment. If the frequency of use of small molecules is increased, there is a high likelihood of causing adverse reactions in other tissues. Thus, it appears that small molecule topical drugs have a greater need to limit the rate of transdermal absorption. Mineral-based materials, such as apatite, diamond and montmorillonite clay, are currently important in limiting the transdermal penetration of bioactive composition of drugs and maintaining local action (Choimet et al., 2020; Shapira et al., 2022; Wang and Phillips, 2022). However, there are fewer studies of mineral-based materials in topical drug delivery systems, and the mechanism of action is unclear. Research on mineral-based materials as topical drug carriers is a blue ocean and more researchers need to participate in it.

Topical pharmaceutical preparations are emerging as an attractive alternative and a rapidly growing market. A variety of factors can influence the final efficacy. No one topical drug or topical drug delivery system is right for everyone. Individualized medication is an important way to improve the effectiveness of drug use. Individualized precision medication administration also achieves optimal outcomes for patients in a more cost-effective manner and reduces unnecessary capital expenditure. However, the complexity of influencing factors and mechanisms of action yet to be fully explored limit the practical application of precise individual administration of topical drugs. Continuing developments in pharmacogenomics, microbiomics and artificial intelligence modelling are continuing to fill the gaps in theoretical research. However, there is still a long way to go for the application of precise individual medication, and it still requires the continuous joint efforts of all parties.

## Figures and Tables

**Table:** 

**CD8^+^**	cluster of differentiation 8^+^
**CD4**	cluster of differentiation 4
**CD14**	cluster of differentiation 14
**Th17**	T helper 17
**Th22**	T helper 22
**IL33**	interleukin-33
**IL4**	interleukin-4
**IL31**	interleukin-31
**Th2**	T helper 2 cell
**ILC3**	Group 3 innate lymphoid cell
**IL-17**	interleukin-17
**IL-22**	interleukin-22
**IL-23**	interleukin-23
**ILC2**	innate lymphoid cell 2
**TNF**	tumour necrosis factor
**TSLP**	thymic stromal lymphopoietin
**ILC1**	innate lymphoid cell 1
**CCL5-CCR3**	C-C motif chemokine ligand 5- C-C chemokine receptor 3
**TNFSF9-TNFRSF9**	Recombinant Tumor Necrosis Factor Ligand Superfamily, Member 9-Recombinant Tumor Necrosis Factor Receptor Superfamily, Member 9
**XCL2-XCR1**	Recombinant Chemokine C-Motif Ligand 2-Recombinant Chemokine C-Motif Receptor 1
**IDO1**	indoleamine2,3-dioxygenase1
**STAT1**	Recombinant Signal Transducer and Activator Of Transcription
**HCAR3**	hydroxycarboxylic acid receptor 3
**MHC**	major histocompatibility complex
**CD163**	cluster of differentiation 163
**STAB1**	stabilin 1
**CEPP**	cyclophosphamide-Etoposide-Procarbazine-Prednisone
**CD40L**	cluster of differentiation4L
**CD11a**	cluster of differentiation11a
**CD70**	cluster of differentiation70
**CpG**	cytosine-phosphoric-guanine
**CD8-CCR7**	cluster of differentiation8- C C chemokine receptor type 7
**CD8-NR4A1**	cluster of differentiation 8- Human Nuclear receptor 4A1
**TGF-β3**	transforming growth factor beta receptor 3
**CYP2D6**	cytochrome P450 2D6
**PharmGKB**	Pharmacogenetics and Pharmacogenomics Knowledge Base
**siRNA**	short interfering RNA
**mRNA**	messenger RNA
**MSC**	mesenchymal stem cells
**TNB**	5-thio-2-nitrobenzoic acid
